# Self-reported risk factors for having *Escherichia coli* sequence type 131 or its *H*30 subclone among US Veterans with a clinical *E. coli* isolate

**DOI:** 10.1017/S0950268818003114

**Published:** 2018-12-03

**Authors:** Amee R. Manges, Paul Thuras, Stephen Porter, James R. Johnson

**Affiliations:** 1 School of Population and Public Health, University of British Columbia, Vancouver, Canada; 2 Minneapolis Veterans Health Care System, Minneapolis, Minnesota, USA; 3 Department of Psychiatry, University of Minnesota, Minneapolis, Minnesota, USA; 4 Department of Medicine, University of Minnesota, Minneapolis, Minnesota, USA

**Keywords:** Antimicrobial resistance, epidemiology, *Escherichia coli*, extraintestinal pathogenic *E. coli*, multidrug resistance, ST131

## Abstract

Among 469 US military veterans with an *Escherichia coli* clinical isolate (2012–2013), we explored healthcare and non-healthcare risk factors for having *E. coli* sequence type 131 and its *H*30 subclone (ST131-*H*30). Overall, 66 (14%) isolates were ST131; 51 (77%) of these were ST131-*H*30. After adjustment for healthcare-associated factors, ST131 remained positively associated with medical lines and nursing home residence. After adjustment for environmental factors, ST131 remained associated with wild animal contact (positive), meat consumption (negative) and pet cat exposure (negative). Thus, ST131 was associated predominantly with healthcare-associated exposures, while non-ST131 *E. coli* were associated with some environmental exposures.

*Escherichia coli* sequence type (ST131) is a recently emerged, multidrug-resistant lineage of *E. coli* that has expanded tremendously over the past two decades to become the main cause of extraintestinal *E. coli* infections in humans [1, 2]. Within ST131, the *H*30R subclone predominates and accounts for almost all fluoroquinolone-resistant isolates [3]. Within ST131-*H*30R, the *H*30Rx subclone is additionally associated with the CTX-M-15 extended-spectrum *β*-lactamase, conferring resistance to extended-spectrum cephalosporins. This broad and often unanticipated resistance leads to adverse clinical outcomes [4].

Preventive interventions against these threatening new pathogens are sorely needed, but few modifiable risk factors have been discovered. Many studies have identified clinical and healthcare-associated risk factors for ST131 infection, usually based on data obtained by medical record review. Here, among US military veterans, we sought novel risk factors for having an ST131-H30 clinical isolate by investigating self-reported clinical factors, demographic characteristics and environmental exposures.

## Methods

### Study subjects and *E. coli* isolates

Consecutive clinical *E. coli* isolates were collected from the Minneapolis VA Medical Center (MVAMC; Minneapolis, MN) clinical microbiology laboratory from May 2012 to December 2013. The source patients were sent an invitation to participate in the study and a paper-based questionnaire, which requested demographic and exposure information. Participants returned completed questionnaires by mail. Telephone follow-up was used to facilitate survey completion for some subjects, without knowledge of laboratory testing results. The MVAMC institutional review board (4181-A) approved the study.

### Clinical microbiology

The MVAMC clinical laboratory identified the *E. coli* isolates using standard methods and determined susceptibility to 12 antimicrobial agents and extended-spectrum beta-lactamase (ESBL) production using the BD Phoenix Automated Microbiology System (Becton Dickinson and Company, Sparks, MD, USA) per manufacturer guidelines, utilising NMIC/ID-123 panels. Minimum inhibitory concentration (MIC) results were interpreted according to Clinical Laboratory and Standards Institute criteria [5].

### Molecular typing

Isolates were characterised using established polymerse chain reaction (PCR)-based assays for membership in the ST131-*H*30 clonal subset or its ST131-*H*30Rx sublineage [3] and O type (O16 and O25b only) [6]. PCR was done using boiled lysates for template DNA, with appropriate positive and negative control strains.

### Statistical analysis

Comparisons of proportions were made using *χ*^2^ and Fisher exact tests. Exposures associated significantly with either ST131 or *H*30 were included in multiple logistic regression models, which were performed separately for clinical and environmental risk factors.

## Results

### Clinical and epidemiological data

Over the 20-month study period (May 2012–December 2013), the MVAMC clinical microbiology laboratory recovered *E. coli* from 925 unique veterans, of whom 469 (51%) opted to complete the study survey. Most subjects were male (82%); half were 66 years or older (Table 1). Most were seen in the outpatient setting (384; 83%); the rest (79; 17%) had been hospitalised for 48 h or more. The most common specimen types were urine (366; 78%), wound swab (52; 11%) and blood (15; 3%).

**Table 1.** Characteristics of 469 veterans with an *Escherichia coli* clinical isolate, stratified by ST131 status

	Prevalence of characteristic, no (column %)			
Characteristic	Total (*n* = 469)	ST131 (*n* = 66)	Non-ST131 (*n* = 403)	*P*-value*
Sex (male)	383 (82)	57 (86)	326 (81)	0.32
Age				0.42
⩽35 years	19 (4)	2 (3)	17 (4)	
36–45	8 (2)	0 (0)	8 (2)	
46–55	65 (14)	5 (8)	60 (15)	
56–65	138 (30)	18 (27)	120 (30)	
66–75	108 (23)	20 (30)	88 (22)	
76–85	92 (20)	15 (23)	77 (19)	
⩾86	39 (8)	6 (9)	33 (8)	
Income				0.43
<$25 000	190 (41)	29 (44)	161 (41)	
$25 000–$50 000	164 (35)	23 (35)	141 (36)	
$50 000–$75 000	52 (11)	7 (11)	45 (11)	
$75 000–$100 000	29 (6)	2 (3)	27 (7)	
>$100 000	8 (2)	0 (0)	8 (2)	
Unknown	18 (4)	5 (8)	13 (3)	
Urban/Rural				0.34
Large city (>20 000)	259 (55)	40 (61)	219 (54)	
Medium to small city (5000–20 000)	81 (17)	7 (11)	74 (18)	
Village (<5000)	83 (18)	15 (23)	68 (17)	
Rural	44 (9)	4 (6)	40 (10)	
Other	1 (<1)	0 (0)	1 (1)	
Dwelling				0.5
Apartment	106 (23)	15 (23)	91 (23)	
Private home	326 (70)	48 (74)	278 (70)	
Institution	11 (2)	0 (0)	11 (3)	
Other	22 (5)	2 (3)	20 (5)	
Households with children	44 (9)	4 (6)	62 (13)	0.31
Health status				0.82
Excellent	27 (6)	4 (6)	23 (6)	
Good	167 (36)	26 (39)	141 (35)	
Average	116 (25)	13 (20)	103 (26)	
Fair	97 (21)	13 (20)	84 (21)	
Poor	59 (13)	10 (15)	49 (12)	
Co-morbidities^a^				
Diabetes	142 (30)	21 (32)	121 (30)	0.77
Chronic lung disease	85 (18)	14 (21)	71 (18)	
Chronic heart disease	114 (24)	7 (11)	107 (27)	
Renal disease	48 (10)	10 (15)	38 (9)	
Immunodeficiency	28 (6)	2 (3)	26 (6)	
Chronic liver disease	20 (4)	1 (2)	19 (5)	
Stroke or neurologic disease	51 (11)	9 (14)	42 (10)	
Cancer	140 (30)	23 (35)	117 (29)	

All variables included results for between 462 and 469 subjects.

a Subjects could report more than one co-morbidity.

*P-value corresponds to an overall *χ*^2^ test.

### Antimicrobial susceptibility

Ampicillin was the most commonly detected resistance phenotype (187; 40%), followed by ciprofloxacin (84; 18%), ampicillin-sulbactam (80; 17%) and trimethoprim-sulfamethoxazole (77; 16%). No isolate was carbapenem-resistant. Eighty-five isolates (18%) were resistant to ⩾3 antimicrobial agents and 21 (4%) to ⩾5 antimicrobial agents.

### Prevalence of ST131 and its subsets

ST131 accounted for 66 (14%) of the 469 study isolates. Of the 66 ST131 isolates, 51 (77%) were ST131-*H*30, of which 43 (84%) represented the *H*30R1 subclone and 8 (12%) the ST131-*H*30Rx subclone. Subjects with ST131 *vs.* non-ST131 isolates did not differ significantly on demographic characteristics, including age, dwelling type, health status, or comorbidities (Table 1).

Among the 66 ST131 isolates, all 51 *H*30 isolates, but only 2/15 (13%) non-*H*30 isolates, were fluoroquinolone-resistant (*P*-value < 0.001). Sixty-three (95%) of the ST131 isolates were typed as O25b; 51 (88%) of these were *H*30.

### Clinical factors and ST131 and ST131-H30

Overall, ST131 was significantly associated with exposure to medical lines (within the past month (*P*-value = 0.005) or 6 months (*P*-value < 0.001)), hospitalisation (*P*-value = 0.004), nursing home residence (*P*-value = 0.001), surgery (*P*-value = 0.01) and antimicrobial exposure in the past 6 months (*P*-value = 0.008) (Table 2). ST131 also was over-represented among isolates from non-blood, wound, or urine specimens (*P*-value = 0.01) (Table 2).

**Table 2.** Clinical factors and *E. coli* ST131 carriage, by ciprofloxacin resistance and ST131 *H*30 sub-group

			All isolates (*n* = 469)			Ciprofloxacin-resistant (*n* = 84)			ST131 isolates (*n* = 66)		
			Prevalence, no (column %)			Prevalence, no (column %)			Prevalence, no (column %)		
Clinical factor	Time frame or sample type	Total	ST131 (*n* = 66)	Non-ST131 (*n* = 403)	*P*-value*	ST131 (*n* = 53)	Non-ST131 (*n* = 31)	*P*-value	*H*30 (*n* = 51)	Non-*H*30 (*n* = 15)	*P*-value
Physician visit	1 month	439	62 (94)	377 (95)	0.66	50 (94)	29 (94)	0.88	48 (94)	14 (100)	0.35
	6 months	406	61 (94)	345 (87)	0.11	51 (96)	30 (97)	0.9	49 (96)	12 (86)	0.15
Medical lines	1 month	209	40 (61)	169 (42)	**0.005**	32 (60)	17 (55)	0.62	30 (59)	10 (67)	0.59
	6 months	169	37 (57)	132 (33)	**<0.001**	33 (62)	15 (48)	0.22	31 (61)	6 (43)	0.23
Hospital stay	1 month	160	27 (41)	133 (33)	0.23	24 (45)	13 (42)	0.77	23 (45)	4 (27)	0.2
	6 months	112	25 (38)	87 (22)	**0.004**	22 (42)	10 (32)	0.4	22 (43)	3 (21)	0.14
Nursing home stay	1 month	26	6 (9)	20 (5)	0.17	5 (9)	4 (13)	0.62	5 (10)	1 (7)	0.76
	6 months	29	10 (16)	19 (5)	**0.001**	10 (19)	4 (13)	0.46	10 (20)	0 (0)	0.07
Surgery	1 month	93	13 (20)	80 (20)	0.96	10 (19)	4 (13)	0.48	9 (18)	4 (27)	0.44
	6 months	90	20 (31)	70 (18)	**0.01**	16 (30)	8 (26)	0.67	16 (31)	4 (29)	0.84
Antibiotic use	1 month	125	23 (35)	102 (26)	0.11	20 (38)	14 (47)	0.43	19 (37)	4 (27)	0.45
	6 months	154	31 (48)	123 (31)	**0.008**	29 (55)	17 (55)	0.99	27 (53)	4 (29)	0.11
Acid reducing drug	1 month	183	25 (40)	158 (40)	0.91	20 (39)	17 (57)	0.13	19 (39)	6 (43)	0.78
Immunosuppression	1 month	11	2 (3)	9 (2)	0.68	1 (2)	2 (7)	0.28	1 (2)	1 (7)	0.34
Hospital inpatient	Current	79	12 (18)	67 (17)	0.71	8 (15)	5 (16)	0.93	8 (16)	4 (27)	0.35
Specimen type	Blood	15	2 (3)	13 (3)	0.93	2 (4)	1 (3)	0.9	2 (4)	0 (0)	0.44
	Urine	366	46 (70)	320 (79)	0.08	40 (75)	25 (81)	0.58	38 (75)	8 (53)	0.12
	Wound	52	8 (12)	44 (11)	0.77	4 (8)	1 (3)	0.42	4 (8)	4 (27)	**0.05**
	Other	36	10 (15)	26 (6)	**0.01**	7 (13)	4 (13)	0.97	7 (14)	3 (20)	0.55

*For some variables data were missing for 2–3 subjects. Hypothesis testing with small numbers (cell size <5) was conducted using Fisher's exact test, otherwise, the *P*-value for the *χ*^2^ test is reported. Bold *P*-values reflect statistically significant differences.

By contrast, among ciprofloxacin-resistant isolates, clinical risk factors did not differ significantly between ST131 (*n* = 53) and non-ST131 (*n* = 31) isolates (Table 2). Similarly, among ST131 isolates, the only clinical risk factor that differed *H*30 from non-*H*30 isolates was wound source, which was weakly associated with non-*H*30 isolates (Table 2).

Multiple logistic regression was used to identify clinical variables independently associated with *E. coli* ST131. Overall, after adjustment for variables significantly associated with ST131 in the univariable analyses, only medical line used in the past 6 months (adjusted OR 2.14, 95% CI 1.23–3.74) and nursing home residence (adjusted OR 2.92, 95% CI 1.3–6.76) remained significantly associated with ST131. In univariable analyses, no clinical variable was associated with ST131 status among ciprofloxacin-resistant isolates and only one variable (wound source) was associated with *H*30 status among ST131 isolates (Table 2) and for these subsets, multiple logistic regression was not performed.

### Environmental factors and ST131 and ST131-H30

Overall, ST131 was associated positively with touching wild animals (*P*-value = 0.03) and negatively with meat consumption (*P*-value = 0.01), cat contact (*P*-value = 0.02) and touching animal feces (*P*-value = 0.03) (Table 3). Among subjects with a ciprofloxacin-resistant isolate (*n* = 84), ST131 was associated positively with farm animal contact (*P*-value = 0.05) and touching wild animals (*P*-value = 0.02) and negatively with using city water (*P*-value = 0.05) and cat contact (*P*-value = 0.01). Among subjects with an ST131 isolate (*n* = 66), *H*30 was associated negatively with past-month out-of-state travel (*P*-value = 0.05).

**Table 3.** Environmental factors and *Escherichia coli* ST131 carriage, by ciprofloxacin resistance and ST131-*H*30 sub-group

		All Isolates (*n* = 469) Prevalence, no (column %)			Ciprofloxacin-resistant (*n* = 84) Prevalence, no (column %)			ST131 (*n* = 66) Prevalence, no (column %)		
		*n* (%)	*n* (%)	*P*-value*	*n* (%)	*n* (%)	*P*-value	*n* (%)	*n* (%)	*P*-value
Prepare meals at home				0.53			0.36			0.87
Sometimes or less often	224	34 (52)	190 (47)		27 (51)	19 (61)		26 (51)	8 (53)	
Most days	243	32 (48)	211 (53)		26 (49)	12 (39)		25 (49)	7 (47)	
Eat meat				**0.01**			0.36			0.07
Sometimes or less often	181	35 (53)	146 (36)		26 (49)	12 (39)		24 (47)	11 (73)	
Most days	286	31 (47)	255 (64)		27 (51)	19 (61)		27 (53)	4 (27)	
Eat fresh fruit				0.65			0.45			0.75
Sometimes or less often	203	30 (46)	173 (43)		24 (45)	11 (37)		23 (45)	7 (50)	
Most days	263	35 (54)	228 (57)		29 (55)	19 (63)		28 (55)	7 (50)	
Eat salad/raw vegetables				0.75			0.56			0.16
Sometimes or less often	261	38 (58)	223 (55)		29 (55)	19 (61)		27 (53)	11 (73)	
Most days	207	28 (42)	179 (45)		24 (45)	12 (39)		24 (47)	4 (27)	
Water source										
City water	300	45 (68)	255 (63)	0.44	36 (68)	27 (87)	**0.05**	34 (67)	11 (73)	0.63
Bottled Water	168	24 (36)	144 (36)	0.92	20 (38)	15 (48)	0.34	20 (39)	4 (27)	0.37
Well water	109	14 (21)	95 (24)	0.67	11 (21)	4 (13)	0.37	11 (22)	3 (20)	0.9
Waste system										
City sewer	363	53 (80)	310 (77)	0.54	42 (79)	29 (94)	0.08	40 (78)	13 (87)	0.48
Septic/field	100	13 (20)	87 (22)	0.73	11 (21)	2 (6)	0.08	11 (22)	2 (13)	0.48
Travel										
Trips outside state 1 month	139	15 (23)	124 (32)	0.19	10 (19)	8 (27)	0.43	9 (18)	6 (43)	**0.05**
Trips outside state 6 months	183	26 (41)	157 (40)	0.91	22 (42)	8 (27)	0.16	22 (44)	4 (29)	0.3
Trips outside USA 1 month	7	0 (0)	7 (2)	0.28	0 (0)	1 (3)	0.19	0 (0)	0 (0)	NS
Trips outside USA 6 months	19	3 (5)	16 (4)	0.81	1 (2)	2 (6)	0.28	1 (2)	2 (14)	0.11
Companion animal contact										
Dog	167	26 (39)	141 (35)	0.49	20 (38)	10 (32)	0.61	20 (39)	6 (40)	0.96
Cat	121	9 (14)	112 (28)	**0.02**	8 (15)	12 (39)	**0.01**	8 (16)	1 (7)	0.37
Bird	9	1 (2)	8 (2)	0.8	1 (2)	1 (3)	0.7	1 (2)	0 (0)	1
Any farm animal contact	27	7 (11)	20 (5)	0.07	6 (11)	0 (0)	**0.05**	6 (12)	1 (7)	0.57
Poultry contact	11	3 (5)	8 (2)	0.2	3 (6)	0 (0)	0.18	3 (6)	0 (0)	0.34
Touch pets or farm animals				0.97			0.71			0.71
Sometimes or less often	269	38 (58)	231 (58)		32 (60)	20 (65)		30 (59)	8 (53)	
Most days	198	28 (42)	170 (42)		21 (40)	11 (35)		21 (41)	7 (47)	
Touch wild animals				**0.03**			**0.02**			0.70
Never or rarely	423	55 (83)	368 (92)		45 (85)	31 (100)		43 (84)	12 (80)	
Sometimes	44	11 (17)	33 (8)		8 (15)	0 (0)		8 (16)	3 (20)	
Touch animal feces				**0.03**			0.14			1.0
Sometimes or less often	426	65 (98)	361 (91)		52 (98)	28 (90)		50 (98)	15 (100)	
Most days	38	1 (2)	37 (9)		1 (2)	3 (10)		1 (2)	0 (0)	

*For some variables, data were missing for 2–3 subjects. Hypothesis testing with small numbers (cell size <5) was conducted using Fisher's exact test, otherwise, the *P*-value for the *χ*^2^ test is reported. Bold *P*-values reflect statistically significant differences.

Multiple logistic regression was used to identify environmental variables independently associated with carriage of *E. coli* ST131. After adjustment for variables associated with ST131 in the univariable analyses, contact with wild animals (adjusted OR 2.49, 95% CI 1.16–5.33) remained positively associated and meat consumption (adjusted OR 0.48, 95% CI 0.28–0.82) and pet cat exposure (adjusted OR 0.37, 95% CI 0.17–0.77) remained negatively associated, with ST131. With wild animal contact in the model, contact with animal feces was no longer associated with ST131 (*data not shown*). Multiple logistic regression was not performed for subjects with ciprofloxacin-resistant *E. coli*, all of whom reported contact with wild or companion animals, or for subjects with ST131 since within this subset only one *H*30-associated variable (out-of-state travel) was identified.

## Discussion

Here we assessed self-reported demographics, clinical factors and environmental exposures of veterans with an *E. coli* clinical isolate as predictors of ST131 status (both overall and for fluoroquinolone-resistant isolates) and, among subjects with an ST131 isolate, of *H*30 subclone status. This is one of few studies to examine non-healthcare-associated risk factors for having a clinical isolate from the pandemic ST131 lineage and specifically its *H*30 subclone.

Our findings support two main conclusions. First, most of the ST131-associated clinical factors represent traditionally recognised risk factors for having an antimicrobial-resistant infection. However, with stratification by fluoroquinolone resistance status, the ST131-specific associations disappeared. This suggests that these healthcare exposures are not specific to ST131, but are generic markers for infection with antimicrobial-resistant *E. coli* generally.

Second, a modestly (albeit statistically significantly) greater proportion of subjects with non-ST131 *E. coli*, as opposed to ST131, reported frequent exposure to certain hypothesised environmental risk factors. Specifically, non-ST131 group members reported more frequent meat consumption, cat ownership, city water usage, recent out-of-state travel and touching animal feces. By contrast, ST131 group members reported more contact with wild animals. This suggests that contact with wild animals, or other variables associated with wild animal exposure, predispose specifically to having ST131, as has been suggested by others [7]. Carriage of the ST131-H30 subclone was associated with wound specimens, while non-H30 carriage was associated with past-month out-of-state travel.

The study has several limitations. First, relatively few subjects reported having several of the candidate risk factors (e.g. travel or farm animal exposure), or had ST131-*H*30Rx, limiting statistical power for these variables. Second, all subjects were veterans, limiting generalisability and possibly contributing to our not finding the typical associations of ST131 with older age, comorbidities and overall poor health [8–10]. Third, the requirement for survey completion may have selected for an uncharacteristically healthy and functional subset of veterans. Fourth, risk factors for *E. coli* acquisition or carriage may have occurred earlier in life and hence were unmeasured in this study. Additional limitations include the risks of false positives from multiple testing and of misclassification due to poor subject recall.

Specific pandemic lineages clearly contribute disproportionately to extraintestinal *E. coli* infections, especially if antimicrobial-resistant, making their potential reservoirs and modes of transmission important to understand. In this veteran-based study population, ST131 was associated preferentially with healthcare-associated exposures and wild animal contact, whereas non-ST131 *E. coli* were associated with other environmental exposures.
